# Comprehensive Evaluation of Compliance With NICE Guidelines for Lithium Monitoring at Kilbarrack East CMHT, Dublin: Audit Cycle Overview

**DOI:** 10.1192/bjo.2026.11706

**Published:** 2026-06-30

**Authors:** Umer Saeed Haroon, Talha Rauf, Louisa Okogwu

**Affiliations:** 1 North Dublin Mental Health Services, Dublin, Ireland; 2 RCSI, Dublin, Ireland

## Abstract

**Aims:**

To evaluate compliance of lithium monitoring practices at Kilbarrack East CMHT with NICE guidelines and identify areas for improvement to enhance patient safety.

**Methods:**

This quantitative audit comprised a retrospective initial audit, where data was collected from August 2024 to February 2025 from patients on lithium therapy. Following the initial audit results, which highlighted significant gaps, a multidisciplinary team (MDT) meeting was held to discuss the findings with all clinical stakeholders. Interventions were implemented in March 2025, including a green sticker system for patient identification, a lithium monitoring sheet for tracking assessments, patient monitoring lists to define responsibilities, a lithium blood clinic, and a new Clinical Nurse Specialist reporting system. A prospective follow-up audit was conducted from May to October 2025, with the overall audit concluding in November 2025. Compliance with NICE guidelines was assessed through indicators such as the frequency of lithium level testing as well as renal and thyroid function tests. Statistical analysis was performed to compare compliance rates between the initial and follow-up audits.

**Results:**

The first audit (Cycle 1) revealed that only 12% (3 out of 25) of patients were compliant with all relevant lithium monitoring guidelines. Although all compliant patients had urea and electrolyte (U&E) levels and thyroid function tests conducted, none had calcium levels tested or BMI measured. In the follow-up audit (Cycle 2) in October 2025, the overall compliance improved, with 68% (17 out of 25) compliant, but significant gaps remained; 32% (8 out of 25) did not have their lithium levels tested, and 44% did not undergo U&E or thyroid function tests. Only 8% had calcium levels tested, and no patients had their BMI recorded. These findings indicate that while there was some progress, critical areas still need attention.

**Conclusion:**

The audit cycle demonstrated substantial deficiencies in compliance with NICE guidelines for lithium monitoring at Kilbarrack East CMHT. While some changes led to improvements, significant gaps remain, underscoring the necessity for continuous quality improvement initiatives. A patient education leaflet was introduced after the second audit to enhance adherence to lithium monitoring protocols. Implementing the recommended action plan aims to enhance adherence to monitoring protocols and improve patient safety in lithium therapy management. Regular audits will continue to check progress and make sure guidelines are followed.

